# The first 1001 days: A scoping review of parenting interventions strengthening good enough parenting in parents with intellectual disabilities

**DOI:** 10.1177/17446295231219301

**Published:** 2023-12-05

**Authors:** Annick Zijlstra, Daniek Joosten, Maroesjka van Nieuwenhuijzen, Bram Orobio de Castro

**Affiliations:** Research Institute Child Development and Education, 1234 University of Amsterdam, Amsterdam, The Netherlands; Research Institute Child Development and Education, 1234 University of Amsterdam, Amsterdam, The Netherlands; Research Institute Child Development and Education, 1234 University of Amsterdam, Amsterdam, The Netherlands; Expect Jeugd, Partners voor Jeugd, Amsterdam, The Netherlands; Research Institute Child Development and Education, 1234 University of Amsterdam, Amsterdam, The Netherlands

**Keywords:** parents with intellectual disabilities, preventive parenting interventions, supporting parents, safeguarding children, 1001 days movement

## Abstract

There are concerns about parents’ parenting skills with intellectual disabilities. However, it is reported that parents with intellectual disabilities show good enough parenting if they are supported effectively and in line with their needs. This scoping review identifies and critically evaluates preventive interventions for parents with intellectual disabilities *and* a cumulation of multiple and complex problems that aim to prepare them for good enough parenting. Six interventions were identified, with preliminary to strong indications of effectiveness. Although none of the interventions focused on all conditions of good enough parenting and only one intervention incorporated all seven key elements to effectively work with parents with intellectual disabilities, the limited evidence on the effectiveness of these interventions suggests that significant and societally relevant effects on parents’ knowledge and skills can be attained. This suggests that more comprehensive early preventive interventions with rigorous evaluations can have a significant impact.

Concerns have been reported about whether parents with mild to borderline intellectual disabilities (hereinafter: intellectual disabilities), who have significant limitations in both intellectual functioning and adaptive behaviour (Schalock et al., 2021), will become good enough parents (Llewellyn and Hindmarsh, 2015; Tarleton and Ward, 2007). In general, children of parents with intellectual disabilities are at greater risk of developmental problems (e.g., Emerson and Brigham, 2014; Llewellyn and Hindmarsh, 2015; Schuengel et al., 2017), and of becoming victims of neglect and abuse (e.g., Slayter and Jensen, 2019; Wickstrom et al., 2017). Consequently, these children are overrepresented in child protection services (e.g., McConnell et al., 2021; Powell, 2016). Parents with intellectual disabilities are considered one of the most vulnerable groups of parents (Wilson et al., 2013), given the high rates of out-home-placements within these families and the increased risk of losing custody (e.g., Booth et al., 2005; McConnell et al., 2021). Besides these consequences for children and families, there are also significant societal consequences. For example, the social cost of youth care is estimated at €100,000 per child for the entire period of youth care (Romeo et al., 2006), and more recent figures show that an out-of-home placement under a family supervision order in the Netherlands costs an average of €17,000 per child per year (Harder et al., 2020).

The increased risks of unsafe parenting circumstances for children of parents with intellectual disabilities can reflect the multiple and complex problems that these parents often face. As a group, parents with intellectual disabilities relatively often experience multiple and complex problems, such as being a young and single parent, living in poverty, having poor mental health, having a lack of social supportive networks (e.g., Aunos et al., 2008; Meppelder et al., 2015; Powell et al., 2017; Wade et al., 2008), and having children with special needs, due to their increased risk of inherited intellectual disabilities (McGaw et al., 2010). A cumulation of these problems is already seen during pregnancy, which impacts the child’s safety once the child is born (Zijlstra et al., 2023). For example, when a child is exposed to violence during pregnancy or when financial or housing problems ensue, the parents’ stress levels increase. This can lead to inadequate parenting and negative family functioning, and ultimately to problems in the children’s psychosocial development, such as problems in mental health, school dropout, addiction, and delinquency (Hindmarsh et al., 2017; Murray et al., 2010). Thus, rather than the limited cognitive abilities of parents with intellectual disabilities directly leading to inadequate parenting, it is especially a cumulation of personal and contextual factors that leads to inadequate parenting (Zijlstra et al., 2023). In conclusion, a cumulation of problems in combination with intellectual disabilities tends to spark a vicious cycle of increasing problems and decreasing strengths that jeopardize the prospects for a new-born child to grow up in a safe environment.

There is, however, a growing body of research that shows that many parents with intellectual disabilities are capable of good enough parenting, as long as they are adequately supported in line with their needs (Collings and Llewllyn, 2012; IASSID, 2008; Koolen et al., 2020). The term ‘good enough parenting’ means a stable, caring, and loving ‘parenting’ of the child, which enables the child to develop good enough (Winnicott 1965 in Hoghughi and Speight, 1998). The term implies that a parent does not have to be perfect in raising his or her baby. It is considered neither realistic nor necessary to expect perfection from parents, and such expectancies would undermine the parenting skills of the majority of parents who do actually meet their children’s needs sufficiently (Winnicott 1965 cited in Hoghughi and Speight, 1998). In line with the ‘Best Interest of the Child Model’, fourteen conditions have been suggested by Zijlstra (2012) that need to be met for children to develop optimally (see Table 1).

**Table 1.** Fourteen conditions of good enough parenting from Zijlstra (2012), p.23-37.

Condition	Definition
1. Adequate physical care	Adequate physical care refers to the care for the child’s health and physical well-being by parents or care-providers. They offer the child a place to live, clothing to wear, enough food to eat and (some) personal belongings. There is a family income to provide for all this. In addition, the parents or care-providers are free of worries about providing for the child’s physical well-being.
2. Safe direct environment	A safe direct physical environment offers the child physical protection. This implies the absence of physical danger in the house or neighbourhood in which the child lives. There are no toxics or other threats in the house or neighbourhood. The child is not threatened by abuse of any kind.
3. Affective atmosphere	An affective atmosphere implies that the parents or care-providers of the child offer the child emotional protection, support and understanding. There are bonds of attachment between the parent(s) or care-giver(s) and the child. There is a relationship of mutual affection.
4. Supportive, flexible childrearing structure	A supportive, flexible childrearing structure encompasses several aspects like: - enough daily routine in the child’s life; - encouragement, stimulation, and instruction to the child and the requirement of realistic demands; - rules, limits, instructions, and insight into the arguments for these rules, limits, and instructions; - control of the child’s behaviour; - enough space for the child’s own wishes and thoughts, enough freedom to experiment and to negotiate over what is important to the child; - no more responsibilities than the child is capable of handling (in this way the child learns the consequences of his behaviour within the limits which the parents or care-providers have set).
5. Adequate examples by the parent	The parents or care-providers offer the child the opportunity to incorporate their behaviour, values and cultural norms that are important, now and in the future.
6. Interest	The parents or care-providers show interest in the activities and interests of the child and in his perception of the world.
7. Continuity in upbringing conditions, future perspective	The parents or care-providers care for the child and bring the child up in a way that attachment bonds develop. Basic trust is to be continued by the availability of the parents or care-providers to the child. The child experiences a future perspective.
8. Safe wider physical environment	The neighbourhood the child grows up in is safe, as well as the society the child lives in. Criminality, (civil) wars, natural disasters, infectious diseases etc. do not threaten the development of the child.
9. Respect	The needs, wishes, feelings and desires of the child are taken seriously by the child’s environment and the society the child lives in. There is no discrimination because of background, race or religion.
10. Social network	The child and his family have various sources of support in their environment upon which they can depend.
11. Education	The child receives a suitable education and has the opportunity to develop his personality and talents (e.g. sport or music).
12. Contact with peers	The child has opportunities to have contacts with other children in various situations suitable to his perception of the world and developmental age.
13. Adequate examples in society	The child is in contact with children and adults who are examples for current and future behaviour and who mediate the adaptation of important societal values and norms.
14. Stability in life circumstances, future perspective	The environment in which the child is brought up does not change suddenly and unexpectedly. There is continuity in life circumstances. Significant changes are prepared for and made comprehendible for the child. Persons with whom the child can identify and sources of support are constantly available to the child, as well as the possibility of developing relationships by means of a common language. Society offers the child opportunities and a future perspective.

Good enough parenting, therefore, needs to be assessed from an ecological perspective, in which it is assumed that many people and circumstances interact and influence a child’s development (e.g., the interplay between children and their partners, home and community environments, and family and human service systems) (Choate and Engstrom, 2014; IASSID, 2008). From that perspective, a good enough parent also accepts their limitations and is willing to accept help and support (Choate and Engstrom, 2014).

Theoretically, effective key elements in parenting interventions for parents with intellectual disabilities are well documented. First, as contextual factors can affect parenting skills and interventional outcomes, interventions must pay attention to the context and the multiple and complex problems these families face, so that stress factors can be eliminated and parents will be more likely to accept further help (Hanson et al., 2023; Koolen et al., 2020; Meppelder et al., 2015; Mildon et al., 2008; Milot et al., 2016). Second, it seems important to intervene early, even during pregnancy, to shift away from a crisis-driven model to a more preventive model (e.g., Dodge, 2019; O'Keeffe and O'Hara, 2008; Stewart and MacIntyre, 2017). Third, to promote a working alliance, the attitude and approach of the professionals need to be supportive and tailored to the needs of parents with intellectual disabilities (Augsberger et al., 2021; Hanson et al., 2023; Koolen et al., 2020) to increase parents' trust in support and make it more likely that parents will seek and accept help (Meppelder et al., 2014; Willems et al., 2007). Fourth, the professionals should have knowledge of the children’s rights to safety (UNICEF, 1989), the rights of the parents with intellectual disabilities to raise a child, and the government's duty to provide support to these families (Hanson et al., 2023; United Nations, 2006). Fifth, interventions must be skill-focused and use behavioural teaching strategies, such as modelling, feedback, praise or tangible reinforcement, and task analysis (Feldman, 1994, as confirmed in Feldman and Tahir, 2016). Sixth, interventions must be home-based (Aunos and Pacheco, 2021; Feldman, 1994), long-term, and intensive (Koolen et al., 2020). Finally, interventions must be in partnership with the family and the informal network of the family in order to increase the resilience of the families (Atkin and Stenfert Kroese, 2021; Scheffers et al., 2020).

However, empirically, little is known about the actual effects of such interventions. Although a wide range of preventive parenting interventions is available, parents with intellectual disabilities are generally explicitly excluded from these interventions, both in research and in practice (Glazemakers and Deboutte, 2013; IASSID, 2008), as these general parenting interventions are often not considered appropriate for them. Unfortunately, parenting interventions specifically for parents with intellectual disabilities are scarce (Coren et al., 2018; Hanson et al., 2023). Moreover, previous reviews of the few studies on parenting training for parents with intellectual disabilities (in chronological order: Feldman, 1994; Wade et al., 2008; Coren et al., 2011; Wilson et al., 2013; Knowles et al., 2015; Coren et al., 2018) emphasized that the evidence concerning interventions for this group was too weak to draw strong conclusions about their effects. On the one hand, the studies included in these reviews were hardly comparable: they focused on a variety of skills and differed in content, techniques, and duration (Knowles et al., 2015; Coren et al., 2018). On the other hand, the reviews did not specifically consider the key elements and the effectiveness of *preventive* parenting interventions, including interventions during pregnancy, whereas, especially for new-borns, specific treatment elements play a key role in supporting parents with intellectual disabilities to provide good enough parenting and prevent unsafe circumstances. Additionally, none of the reviews considered the suitability of interventions for parents with intellectual disabilities *and* a cumulation of risk factors leading to multiple and complex problems, while it is seen that this is often the case within these families.

With the present scoping review we address these issues by aiming to identify and critically evaluate preventive interventions from pregnancy up to the first 1001 days for parents with intellectual disabilities *and* a cumulation of multiple and complex problems. The 'first 1000 days', counting from conception to a child's second birthday, are widely recognized as a crucial developmental period that sets the foundation for a child's future well-being (Black et al., 2017; Richter et al., 2017). In this period, the child's brain and cognition develop exceptionally rapidly. While risk factors that emerge during this period can have a significant impact on long-term development, it also provides a critical window of opportunity for interventions that can have a lasting impact on their development (World Health Organization et al., 2018). As parents with intellectual disabilities often experience a cumulation of multiple and complex problems in the first 1000 days, the current research examined:

1. Which preventive interventions during pregnancy and the first 1001 days have been studied for parents with intellectual disabilities *and* a cumulation of multiple and complex problems to prepare them for good enough parenthood?
2. What are the effects of these interventions on good enough parenting?
3. Which key elements have been incorporated into these interventions?

## Method

### Protocol and registration

The method of this scoping review was based on the scoping review approach proposed by Arksey and O'Malley (2005) and reported in line with the Prisma standards (Page et al., 2021). The protocol has been published on Open Science Framework (https://osf.io/s4z92), before data charting was finished on February 22, 2023.

### Eligibility criteria

This review included English language empirical studies that used any design to assess the effectiveness of a preventive intervention (starting in the period of pregnancy up to the first 1001 days) for parents with intellectual disabilities *and* a cumulation of multiple and complex problems published between 1990-2023. The interventions had to focus on one or more components of good enough parenting. No restrictions were placed on study quality or type of design for assessing the effectiveness of an intervention. Papers were excluded if they described a child-focused intervention that did not involve parent training focused on good enough parenting or interventions for children of parents with intellectual disabilities living in foster care. Reviews, meta-analyses, and book chapters were also excluded from the review.

### Search strategy

**Figure 1.**
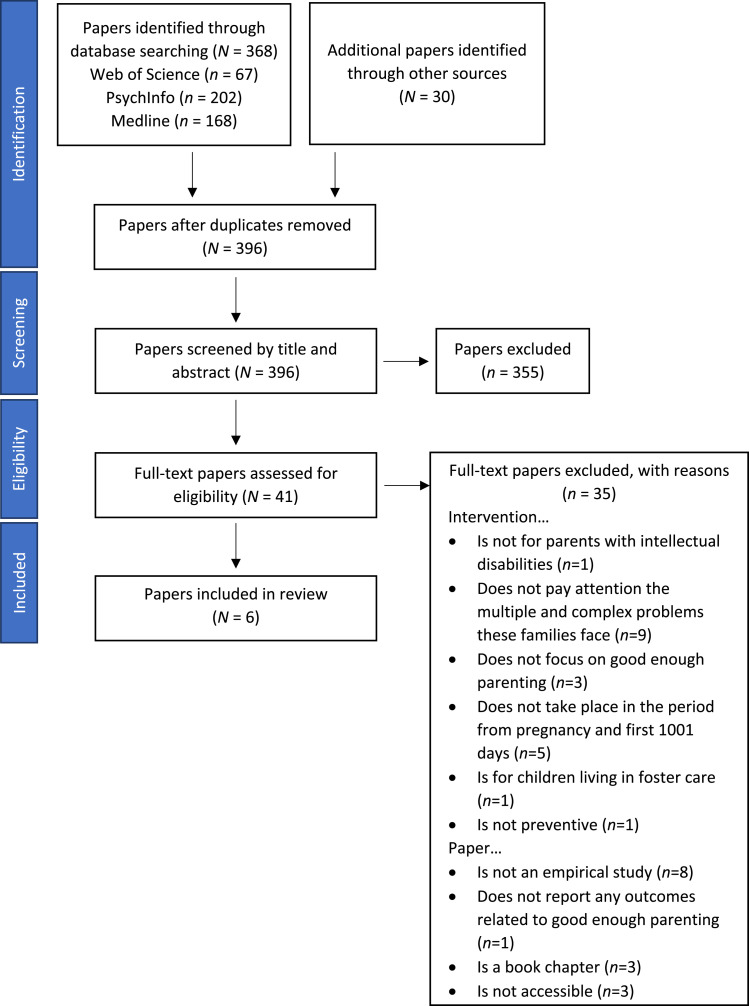
Flowchart of Included and Excluded Papers.

A literature search was carried out in Web of Science, PsycInfo, and Medline databases, using keywords and their synonyms related to the population (e.g., “parents”, “intellectual disab*”), to the intervention period (e.g., “neonatal birth”, “infan*”), and intervention (e.g., “parent training”, “family intervention”) (see the protocol (https://osf.io/s4z92) for the full search strategy). Secondary searches were conducted on references of included papers and relevant reviews and meta-analyses. The 396 identified papers were screened double-blind against the eligibility criteria by two reviewers (AZ and DJ) on title and abstract. Disagreements were resolved by consensus. This procedure resulted in 46 candidate papers. Candidate papers were read in full text and considered for inclusion by one of the two reviewers (AZ or DJ), based on the eligibility criteria. The reviewers double-blindly double-coded 20% of the papers to determine if the paper should be included. Disagreements were resolved by consensus. Eventually, 6 papers met the eligibility criteria. The flowchart is presented in Figure 1.

### Data-charting process

To give an overview of which preventive interventions during pregnancy and the first 1001 days have been studied for parents with intellectual disabilities *and* a cumulation of multiple and complex problems to prepare them for good enough parenthood, papers were coded on publication details (e.g., first author, year of publication, country), specifics of the intervention (e.g., target population, focus, start, duration, intensity, training procedure, and location of the intervention), and characteristics of the study (e.g., research design, participants).

Subsequently, to summarize and to give a critical appraisal of the intervention effects on good enough parenting, papers were coded on effects related to the fourteen conditions of good enough parenting (as presented in Table 1). Study designs were critically evaluated against the Standards of Evidence of Prevention Research (Gottfredson et al., 2015). These standards define three stages of research: efficacy, effectiveness, and scale-up (Gottfredson et al., 2015).

Each stage has its own standards, but the standards are cumulative. Thus, the standards for efficacy are also required for the next stages. Therefore, we first analysed the studies by the efficacy standards, and if they met all standards, we moved on to the following stages. To indicate the strength of the evidence, four different levels of effectiveness have been considered in line with the criteria of Gottfredson et al., (2015): (1) a ‘preliminary first indication of effectiveness’ is indicated when a study using at least a control group or baseline control examined the effectiveness of the intervention; (2) a ‘clear first indication of effectiveness’ is indicated when a study with a randomized controlled trial design with severe limitations or a quasi-experimental study examined the effectiveness of the intervention; (3) a ‘strong indication of effectiveness’ is indicated when a study with a strong research design (e.g., randomized controlled trial) without severe limitations has examined the effectiveness of the intervention; (4) ‘effective’ is indicated when multiple studies with strong research designs (e.g., randomized controlled trials) have examined the effectiveness of the intervention.

Finally, to investigate which key elements have been incorporated into these interventions, the interventions were scored on the seven effective key elements in parenting interventions for parents with intellectual disabilities (e.g., interventions must be skill-focused and use behavioural teaching strategies; interventions must be in partnership with the family and the informal network of the family). The data chart form is presented in our protocol (https://osf.io/s4z92).

## Results

Table 2 summarizes the descriptives of the studies evaluating preventive interventions for parents with intellectual disabilities *and* a cumulation of multiple and complex problems to prepare them for good enough parenting published since 1990, grouped by intervention delivery mode: home-based, and home-based and group-based. For each individual intervention, it provides information on the main focus, the training procedure, and its effects on good enough parenting. In this section, we provide a critical appraisal of each intervention study and its effects on good enough parenting against the Standards of Evidence of Prevention Research (Gottfredson et al., 2015), followed by an evaluation of the incorporated key elements in the interventions.

**Table 2.** Summary of Studies Reviewed.

Name of Intervention	Country	Study reference	The focus of the Intervention	Training procedure	Research Design	Outcomes Related to Good Enough Parenting	Indication of effectiveness
Home-based Parent Training Program	Canada	Feldman et al., (1992a)	Teaching of basic new-born-, infant- and child-care skills.	Home-based weekly training sessions to teach child-care skills, with the use of verbal instructions, specially designed picture books depicting each step of the task analysis, modelling of each step of the task analysis, and feedback on the mother’s actual performance. When the mother performs a task correctly in the next training session, she receives a coupon exchangeable for tangible items. In each session, there is also time to discuss practical issues raised by the mother (e.g., finding a new apartment).	Randomized Controlled Trial	The intervention was effective in teaching child-care skills (e.g., diapering, bathing, feeding, safety) to mothers with low IQ whose children were at risk for child neglect,	Strong indication.
Child-care Training Package	Canada	Feldman et al., (1992b)	Teaching child-care skills.	Weekly training sessions in the mother’s home to teach child-care skills for which the mother had received a mean baseline score below 80%. Training consisted of verbal instructions, modelling, physical guidance, feedback, reinforcement, and if available pictorial manuals. If the mother meets the criterion of 80% correct for a skill, she receives a coupon exchangeable for items. Other topics raised by the mother, can be discussed with the trainer (e.g., finding a job, family issues).	Multiple baseline design across skills and mothers	Child-care skills were improved for all mothers after completing the intervention. The mean percentage of correct performance increased from 58% at baseline to 90% in training. Performance was maintained for 91% in follow-up. Safety steps were performed 100%.	Preliminary first indication.
Public Health Nurse Family Home Visiting	United States	Monsen et al., (2011)	Improve parenting skills.	Weekly to monthly home visits by a public health nurse for 2 years. It is unclear from the paper what the training procedure consists of.	Secondary analysis	Mothers with intellectual disabilities significantly increased their caretaking and parenting knowledge and other improved in other problem areas related to the context they are living in (e.g., income status).	Clear first indication.
Intensive Home-based Parenting Program with a Contextual Fit	Australia	Mildon et al., (2008)	Improving (a) child-care and home-environment; (b) the parent-child interaction; (c) non-corporal strategies to increase child appropriate behaviour.	Weekly home visits for a period of 6 months, focusing on at least one broad goal related to one of the three modules (a, b or c), chosen by the family itself. Skill-focused training was provided with the use of behavioural teaching strategies.	Single group repeated measures design	For all families, there was a significant reduction in parenting hassling’s. The quality of the home environment was only significantly improved for families with children aged 3-6 years old. Parents felt more satisfied and confident in their role as parent after intervention.	Preliminary first indication.
Support to Access Rural Services (STARS)	United States	Keltner et al., (1995)	Improving parent-child interactions.	Weekly group meetings for 1 year, consisting of a conversation about the learning topic, a cooking activity, sharing a meal, and practicing new skills related to the learning topic. Learning topics are generally directly related to parenting, but in some cases learning topics are more contextually related, such as health care or social skills in public spaces. In addition to the group sessions, weekly home visits are planned for reinforcement of the learned skills, and in field trips (e.g., to the public park) the skills can also be practiced. Moreover, the group take tours of common services (e.g., public health clinics) and professionals of public services come to the weekly meetings to tell more about the service.	Randomized controlled trial	After 12 months of intervention, there were still considerable deficiencies in maternal-child interactions for intervention and support groups. However, STARS mothers significantly improved from the pretest to the post-test, compared to the support group. STAR mothers felt more comfortable taking part in activities with their children and gave more positive feedback to their children. STARS mothers more frequently used teaching loops (e.g., alerting the child, providing instructions, allowing sufficient time for performance, and providing feedback).	Strong indication.
Mellow Futures	England/Scotland	Tarleton and Heslop (2021)	Improving parent-child interactions.	The intervention consists of 20-week group sessions, of which 6 weeks are pre-birth and 14 weeks are post-birth. The pre-birth sessions focus on maternal well-being, helping mothers to ‘get to know their unborn baby’ and learning how important warm, positive interactions are to the development of their baby. The post-birth group sessions consist of personal group sessions, in which the mother learns about how her own experiences impact her daily functioning. The rest of the day consists of a joint lunchtime, joint play, and video feedback training, where the focus is on the interaction between the mother and the child. In addition to the group sessions, a specially trained volunteer is available for the mothers on weekly basis for 1-2 hours. The volunteer helps the mother transfer the learned skills in the group to their home setting.	Qualitative interviews; thematic analysis	Out of 30 referrers, 24 reported an improved bonding and interaction between mothers and their babies. Four referrers, however, reported that the intervention did not have any impact on the mother’s ability to care for the baby, as the mother was not able to generalize the learned skills to other situations. For 17 of the 30 babies concerns of child welfare were reduced after the intervention.	Indication of satisfaction and preliminary first indication.

### Home-based interventions

Four preventive home-based interventions for parents with intellectual disabilities *and* a cumulation of multiple and complex problems preparing them for good enough parenthood were identified: the ‘Home Based Parent Training Program’, the ‘Child-care Training Package’, the ‘Home-based Early Intervention’, and the ‘Intensive Home-based Parenting Program with a Contextual Fit’.

The ‘Home-based Parent Training Program’ is focused on teaching basic new-born-, infant-, and child-care skills (e.g., diapering, bathing, and safety) to mothers who are “mentally retarded” (Feldman et al., 1992a: page 16), whose babies (aged 1-23 months) were at risk of child neglect according to social services and child protection agencies. In addition, the intervention also offered advice and support on other issues raised by the mother (e.g., finding a new apartment) (Feldman et al., 1992a). The effectiveness was evaluated in a study by Feldman and colleagues (1992a), using a repeated measure between-group experimental design, in which 22 mothers with intellectual disabilities (mean IQ: 71.6) were compared to 12 mothers without intellectual disabilities.

The mothers with intellectual disabilities were randomly assigned to the intervention group or the waitlist control group, making it a randomized controlled design (RCT) with three groups: intervention group, waitlist intervention group, and control group (without intellectual disabilities). There were significant improvements in parenting behaviour and knowledge of providing adequate physical care. Mothers with intellectual disabilities learned all necessary child-care skills taught in the training (e.g., bathing, feeding) to levels of correct performance seen in a comparison group without intellectual disabilities. Improvements were maintained for up to 76 weeks, although it should be mentioned that there was substantial variability in the duration of the follow-up period (2 to 76 weeks) (Feldman et al., 1992a).

This study indicates a strong indication of the effectiveness of the ‘Home-Based Parent Program’ in improving parents’ knowledge and skills of adequate physical care, a safe direct environment for the child, and stability in life circumstances is indicated. This study is an RCT with three groups and with a follow-up period. However, the results should be interpreted with some caution. In particular, the duration of the follow-up period varied which makes the conclusions about maintenance unclear. Moreover, the parents involved in this study were already in contact with social services and/or child protection agencies because of concerns about the parent’s cognitive abilities in relation to child neglect. This group might, therefore, not be representative of all parents with intellectual disabilities *and* a cumulation of multiple and complex problems. Also, a small sample with only one measurement at the pretest, post-test, and follow-up was used, which limits to test the reliability of the findings.

The ‘Child-care Training Package’ is an intervention focused on teaching basic child-care skills (e.g., bathing, diapering) to mothers with an IQ less than 80 whose young children were at risk for or experiencing neglect, reported by a referrer (e.g., advocate, public health nurse or child welfare worker) (Feldman et al., 1992b). The intervention also addressed other issues raised by the mother (e.g., family issues). The effectiveness of the program was evaluated using a multiple baseline design across skills and across mothers with intellectual disabilities (mean IQ: 74) (Feldman et al., 1992b). There were significant improvements in child-care skills after training, with correct performance of child-care skills increasing from 58% at baseline to 90% in training. Performance was maintained for 91% at follow-up at a mean of 32 weeks and 100% for the safety steps (Feldman et al., 1992b).

This study indicates a preliminary first indication of the effectiveness of the ‘Child-care Training Package’ in improving parents’ knowledge and skills of adequate physical care, providing a safe direct environment for the child, and stability in life circumstances in light of the considerable limitations related to the study design. A single-case experimental design with a small sample size was used, in which there was significant variability between cases in baseline probes. Consequently, it was not possible to have multiple overlapping baseline measurements within and between participants and skills, which made it impossible to have a (complete) baseline comparison period. The pretest-posttest results are therefore hard to interpret and should be interpreted with caution.

‘The Public Health Nurse Family Home Visiting’ is an intervention for first-time, low-income, high-risk pregnant women or new mothers, preparing them to be a parent, with a focus on a healthier pregnancy and birth, learning how to keep children healthy and safe, work on personal and family goals, and connect to programs within their community (Monsen et al., 2011). Although the intervention was not specifically designed for parents with intellectual disabilities, these parents are represented within the target group (Monsen et al., 2011). The feasibility of this intervention for parents with intellectual disabilities was evaluated in a study by Monsen et al. (2011). Based on a secondary analysis of data from clinical documentation, the intervention and outcomes were compared for mothers with intellectual disabilities (based on ‘the cognition problem’, see next paragraph) and a matched cohort comparison group without intellectual disabilities. At the start of the intervention, mothers with intellectual disabilities experienced more often problems within their environment/context (e.g., problems with income or communication with community resources). After the intervention, mothers with intellectual disabilities significantly improved for 7 of 21 problems, versus 10 of 21 problems for mothers without intellectual disabilities, with improvements in caretaking/parenting knowledge and for contextual factors (e.g., income status and knowledge on substance use) for both groups. There were no significant between-group differences in the amount of improvement, which implies that both groups benefit equally from the intervention (Monsen et al., 2011).

A preliminary first indication of the feasibility and effectiveness of the ‘Public Health Nurse Family Home Visiting Program’ for parents with intellectual disabilities to improve their caretaking/parenting knowledge and stability in life circumstances is indicated, given the severe limitations related to the study design of Monsen et al. (2011). It was a pretest-posttest design in which a control group was missing. Moreover, no correction was made for the multiple comparisons between the intervention group of mothers with intellectual disabilities and the matched control group without intellectual disabilities, which could indicate spurious significance. Therefore, it cannot be certainly stated that the effects for parents with intellectual disabilities are related to the intervention or are related to other interventions offered to the parents in the same period. Moreover, it is unclear to what extent this sample of parents with intellectual disabilities was representative of all parents with intellectual disabilities, given that the classification of intellectual disabilities in this study was based on the presence of a ‘cognition problem’, which was defined as “the ability to think and use information” (Monsen et al., 2011: page 486).

Finally, the ‘Intensive Home-based Parenting Program with a Contextual Fit’, is a parenting intervention for parents with an intellectual disability (i.e., “parents were included if they met any of the three following criteria: significantly sub-average general intellectual functioning concurrent with significant deficits in adaptive behaviour (American Association of Mental Retardation 2002); (ii) attended a special education school; or (iii) who had self-identified, or been identified by the referring agency, as having cognitive limitations resulting in difficulties with learning” (Mildon et al., 2008: page 379)) with children aged 6 months through 6 years. The intervention pays specific attention to the context of the family in which the intervention is provided. The parenting program is based on other evidence-based interventions for parents with intellectual disabilities (Feldman 1994, 1998; Tymchuk 1998; Llewellyn et al., 2003), with specific strategies added to tailor the intervention to the contextual problems of these families. The added value of the strategies to fit the intervention to the context of the families was assessed with the use of a single-group repeated measures design with 24 participants with intellectual disabilities (mean IQ: 67.58) by Mildon and colleagues (2008). However, only the contextual fit was repeatedly measured, and the other dependent variables (e.g., parent’s perceived stress and quality of the home environment) were only assessed pretest, post-test, and at three months follow-up. There were small significant reductions in parenting hassles (e.g., cleaning up toys) and small improvements in the quality of the home environment. The improvements were significant for families with children aged 3-6 years, but not for younger children under three years. Parents themselves also experienced a change: they were feeling more confident and satisfied in their role as parents after the intervention. Finally, high levels of satisfaction with the intervention were reported, implying that the contextual approach was helpful.

This study indicates a preliminary first indication of the effectiveness of the ‘Intensive Home-based Parenting Program with a Contextual Fit’ for parents with intellectual disabilities to improve their skills and knowledge on providing adequate physical care, a safe direct physical environment, an affective atmosphere, a supportive, flexible childrearing structure, and stability in life circumstances. The evidence is limited by the single case design with only pretest, post-test and follow-up measurements for the dependent variables. In addition, the sample size was small and the sample was not randomly selected nor matched to a control sample. It is therefore possible that specific characteristics of the sample contributed to the success of the intervention. Moreover, the follow-up period was limited to only three months, meaning that no conclusions can be drawn about the long-term effects of the intervention. Overall, these limitations preclude any firm conclusions that can be made about the effectiveness of the intervention.

### A combination of home-based and group-based interventions

We identified two interventions combining home and group-based sessions for parents with intellectual disabilities *and* a cumulation of multiple and complex problems preparing them for good enough parenthood: ‘Support to Access Rural Services (STARS)’ and ‘Mellow Futures’.

‘STARS’ is a family intervention for mothers from rural areas who are “developmentally disabled and have intellectual limitations” (IQ less than 85) (Keltner et al., 1995: page 38) and have children aged 1-4 years. The intervention consists of weekly small-group meetings and home visits by a social worker. The intervention is focused on improving parent-child interactions and has a broad focus on the circumstances families live in (e.g., by inviting social services to the group meetings to explain more about how to use the service). ‘STARS’ is the only intervention that involves the social network of the family, inviting them to meetings to support parents in knowledge and skill acquisition (Keltner et al.,1995). The effectiveness of ‘STARS’ was assessed in an RCT by Keltner and colleagues (1995), in which 40 mothers were randomly assigned to the intervention group or a support group. After 12 months of ‘STARS’, there were still considerable deficiencies in mother-child interactions for both the intervention group and the support group. Nonetheless, the difference in the range of improvement was only significant for parents who participated in ‘STARS’, indicating the effectiveness of STARS for overall maternal-child interactions. Moreover, at the end of the study, parents who participated in ‘STARS’ reported that they felt more comfortable taking part in activities with their children and gave more positive feedback. They developed so-called teaching loops: alerting the child, providing instruction to the child, allowing sufficient time for performance, and providing feedback to the child (Keltner et al., 1995).

This study indicates a strong indication of the effectiveness of ‘STARS’ for parents who are developmentally disabled and have intellectual limitations in rural areas in the USA to improve the affective atmosphere in which the parent-child interaction takes place, and to improve a supportive, flexible childrearing structure. The indication for effectiveness is strong: a randomized controlled trial without severe limitations other than a modest sample size was conducted. Other minor limitations were that the study sample was a very specific population of women with very low IQ (mean IQ intervention group: 59) and from rural areas, therefore it is unclear to what extent the results can be generalized to other families. Moreover, it was not assessed to what extent the learned skills were maintained over a longer period of time.

‘Mellow Futures’ is another intervention that combines group-based and home-based sessions. The intervention aims to create positive parent-child interactions in expectant parents with “borderline/milder learning disabilities” (IQ above 70) (Tarleton and Heslop, 2021: page 1276), rather than strengthening parenting skills. In addition, the intervention also focuses on the context in which the mother grew up and the current context, and how this affects the relationship between parent and child (Tarleton and Heslop, 2021). The intervention was evaluated with the use of a qualitative study design by Tarleton and Heslop (2021), in which 30 mothers and 31 professionals who referred the mothers to the program were interviewed at the start and end of the intervention. Thematic analysis showed a generally positive impact of the program on bonding and interaction between mother and child. Also, for 17 of the 30 babies, concerns about child welfare were reduced after the intervention, and in one case the program helped the mother realize that she was not able to take care of the baby, which can also be seen as a positive result. However, there were four referrers (13.3%) who stated that the intervention did not have any impact on the mother’s ability to care for their baby, as mothers were not able to integrate the content into their lives (Tarleton and Heslop, 2021).

This study demonstrates program satisfaction in participants, and despite the qualitative study design, it does indicate a preliminary first indication of effectiveness, because the study investigated the change in parenting over time. Preliminary first indications of the effectiveness of ‘Mellow Futures’ were found for improving parents’ skills and knowledge in providing an affective atmosphere, a supportive, flexible childrearing structure, stability in life circumstances, and strengthening the social network of the family. However, the results should be interpreted with caution, given that the claims of the authors on the success of the intervention in helping expectant mothers with intellectual disabilities to take care of their children are thus based on the opinion and experiences of those who actually take part in the intervention. No valid measures of child functioning or parenting, nor an experimental design was used to test the actual effectiveness of the intervention. Moreover, it is not clear whether the positive experiences were due to the intervention itself because some mothers also received other services next to the intervention.

### Incorporated key elements

Several effective elements are known that may strengthen good enough parenting in parents with intellectual disabilities, as discussed earlier in this review. We examined which of these elements were used in the interventions we reviewed. ‘STARS’ was the only intervention that incorporated all key elements. However, based on the papers, it was sometimes unclear whether certain key elements have been incorporated in the interventions, specifically for the key element of knowledge on the legal framework of parenting with intellectual disabilities. Also, none of the papers examined whether the intervention outcomes were due to the incorporation of certain key elements.

All interventions included in this review, were *preventive*, meaning that they started between pregnancy and the first 1001 days, although only ‘The Public Health Nurse Family Home Visiting’ and ‘Mellow Futures’ started during pregnancy. For most interventions, it was described that a *good working alliance* between professional and family was highly valued (‘The Public Health Nurse Family Home Visiting’, ‘Intensive Home-based Parent Program with a Contextual Fit’, ‘STARS’, ‘Mellow Futures’). For the ‘Home-based Parent Program’ and the ‘Child-care Training Package’, it was not clear from the paper to what extent a good working alliance was a key element in the intervention. This, however, does not mean that a good working alliance was not considered important within these interventions. The same applies to the key element *knowledge of the legal framework on parenting with intellectual disabilities*. Only for ‘STARS’ it was specifically mentioned that the professionals had to have knowledge of the legal framework of parenting with intellectual disabilities. However, most interventions did mention it (implicitly or explicitly) or referred to the duty to provide support for parents to raise their children. For example, most parents in the interventions were referred to the intervention by community professionals because of concern about mothers’ abilities to adequately care for their children. All interventions were *skill-focused and made use of behavioural teaching strategies*. Also, all interventions were (partly) *home-based* and were *intensive* (at least 1 visit a week). However, only ‘The Public Health Nurse Family Home Visiting’ and ‘STARS’ were *long-term*, meaning lasting longer than 1 year. Finally, from what was described in the papers, only ‘STARS’ was *in partnership with the social network of the family*, by inviting the social network to group meetings. For example, when there were educational sessions about preventable diseases (e.g., AIDS), family members were invited to support the parents with intellectual disabilities and gain knowledge together.

## Overall conclusion

The aim of this scoping review was to identify and critically evaluate preventive interventions for parents with intellectual disabilities *and* a cumulation of multiple and complex problems preparing them for good enough parenthood in the first 1001 days. It is important to acknowledge that this review only considered interventions that were examined for their effectiveness and that this review thus does not provide an overview of all existing interventions for parents with intellectual disabilities *and* a cumulation of multiple and complex problems preparing them for good enough parenthood. In this scoping review, we identified six different interventions, which focused on only 6 of the 14 components of good enough parenting (as defined by Zijlstra, 2012). These interventions were limited in research on the effectiveness since none of the studies fulfilled all requirements of study quality. Moreover, in most interventions, not all key elements important for providing effective parenting support to parents with intellectual disabilities were incorporated. However, the limited evidence suggests that even when addressing only part of the components of good enough parenting and with only part of the key elements, significant and societally relevant effects on parenting skills in parents with intellectual disabilities *and* a cumulation of multiple and complex problems can be attained, with positive consequences for child development and well-being. This suggests that more comprehensive early preventive interventions with rigorous evaluations can have a major impact.

Concerning components of good enough parenting, it is striking that the focus of most interventions is on conditions within the family and is less focused on conditions in society. This may have to do with the fact that some conditions in society are not yet a problem for these families, given the developmental phase the young child is in, such as contact with peers, and education. Another possible explanation is that the conditions in society cannot be effectively addressed through parenting interventions. Nonetheless, from the ecological perspective of child development, it is important to consider the societal component in parenting interventions as well, given that the child’s development is also influenced by environmental factors in society (Bronfenbrenner, 1979; Zijlstra, 2012). Inadequate quality of only one of those conditions can already lead to damage to the quality of other conditions. For example, if a mother is behind in rent payments and worries about an eviction, the affective atmosphere is threatened because the mother is not able to be in a positive interaction with her child anymore. Subsequently, this can affect societal conditions, for example, because children have not learned positive interactions, they may experience problems in contact with peers, and there may also be a greater risk that these children will experience their own financial problems later in life (Hubler et al., 2016; McConnell et al., 2011; Zijlstra, 2012). Therefore, it is important that interventions are responsive to all fourteen conditions of good enough parenting, to provide a context in which children can develop optimally. Currently, there is no such intervention for parents with intellectual disabilities *and* a cumulation of multiple and complex problems that have been tested for effectiveness.

In this review, we critically evaluated the effectiveness of the interventions. There were indicators that all interventions reviewed were somewhat effective in promoting good enough parenthood. With the exception of the ‘Home-Based Parent Training Program’ (Feldman et al., 1992a) and ‘STARS’ (Keltner et al., 1995), the strength of the evidence was, however, relatively weak according to the Standards of Evidence for efficacy/effectiveness study (Gottfredson et al., 2015). Moreover, none of the intervention descriptions fulfilled all requirements for an efficacy trial, and therefore the requirements for effectiveness and scale-up were not even assessed. Nevertheless, major limitations related to the study designs also resulted in the Standards of Evidence criteria not being met. For example, the use of small sample sizes, qualitative measures, and in the case of (small) RCTs non-validated measurements, unclear descriptions of the randomization process, and non or small follow-up periods. For follow-up research, we strongly recommend conducting multiple effectiveness studies on the same intervention with strong research designs and follow-up periods of at least six months. The research design is preferably an RCT, but in some cases, this is neither feasible nor always the “golden standard”. Although opinions still vary greatly on this, we advise, in line with the guidelines of Gottfredson et al., (2015), that there should be at least a control condition that did not receive the intervention. Preferably, bias should be minimized by randomization to the conditions, but as said, in some cases, randomization is not practicable or possible. Comparison series design can provide a solution in this case.

However, our critical evaluation may have been rather strict, given that the standards are relatively new. The interventions included in this review, cover a time period from 1992 till 2021. This period witnessed a shift in research methodologies and it was only in 2005 that the first set of Standards of Evidence were published (Flay et al., 2005). For example, while RCTs were less common in the 1990s, they are now the “golden standard”. Moreover, as the authors of the Standards of Evidence underline, it will take years before researchers incorporate these standards into their research (Gottfredson et al., 2015). A great number of preventive research does not yet follow the research cycle at all. For example, the effects of a widely implemented intervention have been evaluated (scale-up), while a randomized efficacy trial has not been done yet (efficacy) (Gottfredson et al., 2015). Nevertheless, due to the lack of solid research, no firm conclusions about the effectiveness of the interventions can be made.

Additionally, we evaluated whether the interventions incorporated the key elements to effectively work with parents with intellectual disabilities. It seemed that only ‘STARS’ incorporated all key elements, while we know from the literature that these key elements are important for providing good support to parents with intellectual disabilities aimed at strengthening good enough parenting. The interventions reviewed are often of short duration, while systematic reviews underline that families with intellectual disabilities *and* a cumulation of multiple and complex problems need long-term support (Holwerda et al., 2014). Unfortunately, it is the case that professionals often lack time, training, and material resources to effectively work with these parents. This emphasizes the absence, failure, or unavailability of adequate parenting support for parents with intellectual disabilities (Castell and Stenfert Kroese, 2016; Glazemakers and Deboutte, 2013; Tarleton and Ward, 2007). Moreover, from the information in the papers, it was often unclear whether certain key elements have been incorporated in the interventions, specifically for the key elements of good working alliance and knowledge on the legal framework of parenting with intellectual disabilities. Considering the Standards of Evidence, “the intervention must be described at a level that would allow others to implement/replicate it” (Gottfredson et al., 2015: page 5), which is thus not the case for most interventions reviewed. For future studies on the effectiveness of parenting intervention for parents with intellectual disabilities *and* a cumulation of multiple and complex problems, it is advised to describe all key elements incorporated, even if only in supplementary materials. Based on the information available in the papers of the interventions reviewed, we can only conclude that not all key elements were incorporated which are important in working with parents with intellectual disabilities *and* a cumulation of multiple and complex problems.

In conclusion, this review has highlighted that there is a lack of proven preventive *effective* interventions to support good enough parenting in expectant parents with intellectual disabilities *and* a cumulation of multiple and complex problems. Existing interventions do not focus on all fourteen conditions of good enough parenting, do not include all key elements that are important to support these families effectively, and the effectiveness of the current interventions is limited. Although the knowledge is available about what is needed to effectively support these parents in overcoming unsafe parenting conditions for their new-born child, this does not yet seem to be implemented in the interventions reviewed.

However, parents with intellectual disabilities have the right to adequate and effective support in raising their children (United Nations, 2006). Furthermore, the worldwide initiative to invest in early childhood development reinforces the significance of providing preventive care to vulnerable populations during the crucial first 1000 days (World Health Organization et al., 2018). To effectively support parents with intellectual disabilities *and* a cumulation of multiple and complex problems in the first 1000 days, we strongly recommend implementing a youth care system in which preventive continuous sustainable support can be provided to families with intellectual disabilities. This preventive support must include the seven key elements as described in the introduction to effectively support these parents, which can be intensified when needed, and reduced when possible. In doing so, professionals should be supported with training and supervision in working with parents with intellectual disabilities to fit the needs of these families. Moreover, even if professionals are well trained, the child welfare system fails. Professionals often lack the time, finances, and appropriate materials to offer adequate support (MacIntyre et al., 2019; Pytlowana and Stenfert Kroese, 2021). Therefore, governmental policy should be more aware of the needs of these families and the professionals who support them.

Regardless of the constraints of the currently available interventions and their effectiveness, the existing interventions seem to have significant and societally relevant effects on parenting skills in parents with intellectual disabilities *and* a cumulation of multiple and complex problems, with positive effects on child development and well-being. This suggests that major impacts can be achieved with more comprehensive interventions. We, therefore, recommend investing in the (further) development and evaluation of preventive interventions for parents with intellectual disabilities *and* a cumulation of multiple and complex problems focusing on good enough parenting in the first 1001 days, to shift away from a crisis-driven model towards a preventive model, in which unsafe parenting conditions for the new-born will be prevented and high societal costs will be reduced.

## References

[bibr1-17446295231219301] Arksey H O’Malley L (2005) Scoping studies: towards a methodological framework. International Journal of Social Research Methodology 8: 19−32.

[bibr2-17446295231219301] Atkin C Stenfert Kroese B (2021) Exploring the experiences of independent advocates and parents with intellectual disabilities, following their involvement in child protection proceedings. Disability & Society 37(9): 1456-1478. DOI: 10.1080/09687599.2021.1881884

[bibr3-17446295231219301] Augsberger A Oorsouw WV Verharen L et al. (2021) Supporting parents with intellectual disabilities in child welfare: A systematic review. Journal of Applied Research in Intellectual Disabilities 34: 1284-1285.

[bibr4-17446295231219301] Aunos M Feldman M Goupil G , et al. (2008) Mothering with intellectual disabilities: relationship between social support, health and well-being, parenting and child behavior outcomes. Journal of Applied Research in Intellectual Disabilities 21(4): 320-330. DOI: 10.1111/j.1468-3148.2008.00447.x

[bibr5-17446295231219301] Aunos M Pacheco L (2021) Able or unable: how do professionals determine the parenting capacity of mothers with intellectual disabilities. Journal of Public Child Welfare 15(3): 357-383. DOI: 10.1080/15548732.2020.1729923

[bibr6-17446295231219301] Black MM Walker SP Fernald LCH , et al. (2017) Early childhood development coming of age: science through the life course. Lancet 389 (10064): 77–90. DOI: 10.1016/S0140-6736(16)31389-7 27717614 PMC5884058

[bibr7-17446295231219301] Booth T Booth W McConnell D (2005) The prevalence and outcomes of care proceedings involving parents with learning difficulties in the family courts. Journal of Applied Research in Intellectual Disabilities 18(1): 7-17. DOI: 10.1111/j.1468-3148.2004.00204.x

[bibr8-17446295231219301] Bronfenbrenner U (1979) Contexts of child rearing: Problems and prospects. American Psychologist 34(10): 844–850. DOI: 10.1037/0003-066X.34.10.844

[bibr9-17446295231219301] Castell E Stenfert Kroese B (2016) Midwives' experiences of caring for women with learning disabilities: A qualitative study. Midwifery 36: 35-42. DOI:10.1016/j.midw.2016.02.001 27106942

[bibr10-17446295231219301] Choate PW Engstrom S (2014) The “good enough” parent: Implications for child protection. Child Care in Practice 20(4): 368-382. DOI: 10.1080/13575279.2014.915794

[bibr11-17446295231219301] Collings S Llewellyn G (2012) Children of parents with intellectual disability: Facing poor outcomes or faring okay? Journal of Intellectual & Developmental Disability 37(1): 65-82. DOI: 10.3109/13668250.2011.648610 22300240

[bibr12-17446295231219301] Coren E Thomae M Hutchfield J (2011) Parenting training for intellectually disabled parents: A Cochrane systematic review. Research on Social Work Practice 21(4): 432-441. DOI: 10.1177/1049731511399586

[bibr13-17446295231219301] Coren E Ramsbotham K Gschwandtner M (2018) Parent training interventions for parents with intellectual disability. Cochrane Database of Systematic Reviews (7): DOI: 10.1002/14651858.CD007987.pub3 PMC6513025 30004571

[bibr14-17446295231219301] Dodge KA (2019) Redefining the science and policy of early childhood intervention programs. Paediatrics 144(6). DOI: 10.1542/peds.2019-2606 31748252

[bibr15-17446295231219301] Emerson E Brigham P (2014) The developmental health of children of parents with intellectual disabilities: Cross sectional study. Research in Developmental Disabilities 35(4): 917-921. DOI: 10.1016/j.ridd.2014.01.006 24485469

[bibr16-17446295231219301] Feldman MA (1994) Parenting education for parents with intellectual disabilities: A review of outcome studies. Research in Developmental Disabilities 15(4): 299-332. DOI: 10.1016/0891-4222(94)90009-4 7972968

[bibr17-17446295231219301] Feldman MA Tahir M (2016) Skills training for parents with intellectual disabilities*.* In: Singh NN (eds) Handbook of evidence-based practices in intellectual and developmental disabilities. Cham: Springer, pp.615-631.

[bibr18-17446295231219301] Feldman MA Case L Garrick M MacIntyre-Grande W Carnwell J Sparks B (1992b) Teaching child‐care skills to mothers with developmental disabilities. Journal of Applied Behavior Analysis 25(1): 205-215. DOI:10.1080/13668259900033861 1582966 PMC1279667

[bibr19-17446295231219301] Feldman MA Case L Sparks B (1992a) Effectiveness of a child-care training program for parents at-risk for child neglect. Canadian Journal of Behavioural Science/Revue anadienne des sciences du comportement 24(1): 14. DOI: 10.1037/h0078698

[bibr20-17446295231219301] Feldman M (1998) Parents with intellectual disabilities: implications and interventions. In: Lutzker J (ed) Handbook of Child Abuse Research and Treatment. New York: Plenum press, pp.401-419.

[bibr21-17446295231219301] Flay BR Biglan A Boruch RF , et al. (2005) Standards of evidence: criteria for efficacy, effectiveness and dissemination. Prev Sci 6(3): 151-175. DOI: 10.1007/s11121-005-5553-y 16365954

[bibr22-17446295231219301] Glazemakers I Deboutte D (2013) Modifying the ‘Positive Parenting Program’ for parents with intellectual disabilities. Journal of Intellectual Disability Research 57(7): 616-626. DOI: 10.1111/j.1365-2788.2012.01566.x 22554356

[bibr23-17446295231219301] Gottfredson DC Cook TD Gardner FEM. (2015) Standards of Evidence for Efficacy, Effectiveness, and Scale-up Research in Prevention Science: Next Generation. Prevention Science 16(7): 893-926. DOI: 10.1007/s11121-015-0555-x 25846268 PMC4579256

[bibr24-17446295231219301] Hanson SL Cook TD Gardner FEM. (2023) Guidelines for assessment and intervention with persons with disabilities: An Executive Summary. American Psychologist*.* Online ahead of print 16 March 2023. DOI: 10.1037/amp0001150 36931825

[bibr25-17446295231219301] Harder A Knorth E Kuiper C (eds) (2020) Uithuisgeplaatste jeugdigen: Sleutels tot succes in behandeling en onderwijs. Amsterdam: SWP Uitgeverij.

[bibr26-17446295231219301] Hindmarsh G Llewellyn G Emerson E (2017) The social‐emotional well‐being of children of mothers with intellectual impairment: a population‐based analysis. Journal of Applied Research in Intellectual Disabilities 30(3): 469-481. DOI: 10.1111/jar.12306 27878941

[bibr27-17446295231219301] Hoghughi M Speight ANP (1998) Good enough parenting for all children—a strategy for a healthier society. Archives of disease in childhood 78(4): 293-296. 9623389 10.1136/adc.78.4.293 PMC1717536

[bibr28-17446295231219301] Holwerda A Jansen D Reijneveld M (2014) De effectiviteit van hulpverlening aan multiprobleemgezinnen: Een overzicht. Groningen: Universitair Medisch Centrum Groningen.

[bibr29-17446295231219301] Hubler DS Burr BK Gardner BC , et al. (2016) The Intergenerational Transmission of Financial Stress and Relationship Outcomes. Marriage & Family Review 52(4): 373-391. DOI: 10.1080/01494929.2015.1100695

[bibr30-17446295231219301] IASSID Special Interest Research Group on Parents and Parenting with Intellectual Disabilities (2008) Parents labelled with Intellectual Disability: Position of the IASSID SIRG on Parents and Parenting with Intellectual Disabilities. Journal of Applied Research in Intellectual Disabilities 21(4): 296-307. DOI: 10.1111/j.1468-3148.2008.00435.x

[bibr31-17446295231219301] Keltner B Finn D Schearer D (1995) Effects of family intervention on maternal-child interaction for mothers with developmental disabilities. Family and Community Health: 35-49.

[bibr32-17446295231219301] Knowles C Machalicek W Van Norman R (2015) Parent education for adults with intellectual disability: A review and suggestions for future research. Developmental neurorehabilitation 18(5): 336-348. DOI: 10.3109/17518423.2013.832432 24088181

[bibr33-17446295231219301] Koolen J van Oorsouw W Verharen L Embregts P (2020) Support needs of parents with intellectual disabilities: Systematic review on the perceptions of parents and professionals. Journal of Intellectual Disabilities 24(4): 559-583. DOI: 10.1177/1744629519829965 30832525

[bibr34-17446295231219301] Llewellyn G McConnell D Honey A Mayes R Russo D (2003) Promoting health and home safety for children of parents with intellectual disability: a randomized controlled trial. Research in Developmental Disabilities 24(6): 405–431. DOI: 10.1016/j.ridd.2003.06.001 14622893

[bibr35-17446295231219301] Llewellyn G Hindmarsh G (2015) Parents with intellectual disability in a population context. Current Developmental Disorders Reports 2(2): 119-126. DOI: 10.1007/s40474-015-0042-x 25938007 PMC4408356

[bibr36-17446295231219301] MacIntyre G Stewart A McGregor S (2019) The double-edged sword of vulnerability: Explaining the persistent challenges for practitioners in supporting parents with intellectual disabilities. Journal of Applied Research in Intellectual Disabilities 32(6): 1523-1534. DOI: 10.1111/jar.12647 31318123

[bibr37-17446295231219301] McConnell D Aunos M Pacheco L Feldman M (2021) Child maltreatment investigations in Canada. Main and moderating effects of primary caregiver cognitive impairment. Child Maltreatment 26(1): 115-125. DOI: 10.1177/1077559520910806 32228189

[bibr38-17446295231219301] McConnell D Breitkreuz R Savage A (2011) From financial hardship to child difficulties: main and moderating effects of perceived social support. Child: care, health and development 37: 679-691. DOI: 10.1111/j.1365-2214.2010.01185.x 21143271

[bibr39-17446295231219301] McGaw S Scully T Pritchard C (2010) Predicting the unpredictable? Identifying high-risk versus low-risk parents with intellectual disabilities. Child Abuse & Neglect 34(9): 699-710. DOI: 10.1016/j.chiabu.2010.02.006 20674975

[bibr40-17446295231219301] Meppelder M Hodes M Kef S Schuengel C (2014) Parents with intellectual disabilities seeking professional parenting support: The role of working alliance, stress and informal support. Child Abuse & Neglect 38(9): 1478-1486. DOI: 10.1016/j.chiabu.2014.04.006 24856130

[bibr41-17446295231219301] Meppelder M Hodes M Kef S Schuengel C (2015) Parenting stress and child behavior problems among parents with intellectual disabilities: the buffering role of resources. Journal of Intellectual Disability Research 59(7): 664-667. DOI: 10.1111/jir.12170 25472805

[bibr42-17446295231219301] Mildon R Wade C Matthews J (2008) Considering the contextual fit of an intervention for families headed by parents with an intellectual disability: An exploratory study. Journal of Applied Research in Intellectual Disabilities 21: 377-387. DOI: 10.1111/j.1468-3148.2008.00451.x

[bibr43-17446295231219301] Milot E Turcotte D Tétreault S (2016) Support to parents with cognitive limitations: parental abilities and social participation. British Journal of Learning Disabilities 44(1): 71-77. DOI: 10.1111/bld.12113

[bibr44-17446295231219301] Monsen K Sanders A Yu F Radosevich D Geppert J (2011) Family home visiting outcomes for mothers with and without intellectual disabilities. Journal of Intellectual Disabilities Research 55(5): 484-499. DOI: 10.1111/j.1365-2788.2011.01402.x 21366756

[bibr45-17446295231219301] Murray J Irving B Farrington DP Colman I Bloxsom CAJ (2010) Very early predictors of conduct problems and crime: Results from a national cohort study. The Journal of Child Psychology and Psychiatry 51(11): 1198-1207. DOI: 10.1111/j.1469-7610.2010.02287.x 20633069

[bibr46-17446295231219301] O’Keeffe N O’Hara J (2008) Mental health needs of parents with intellectual disabilities. Current Opinion in Psychiatry, 21(5), 463-468. DOI: 10.1097/YCO.0b013e328305e61f 18650688

[bibr47-17446295231219301] Page MJ McKenzie JE Bossuyt PM , et al. (2021) The PRISMA 2020 statement: an updated guideline for reporting systematic reviews. BMJ, 372(71): 1-9. DOI:10.1136/bmj.n71 PMC8005924 33782057

[bibr48-17446295231219301] Powell RM Parish SL Akobirshoev I (2016) Health of young children whose mothers have intellectual disability. American journal on intellectual and developmental disabilities 121(4): 281-294. DOI: 10.1352/1944-7558-121.4.281 27351697 PMC9402285

[bibr49-17446295231219301] Powell RM Parish SL Akobirshoev I (2017) The health and economic well-being of US mothers with intellectual impairments. Journal of Applied Research in Intellectual Disabilities 30(3): 456-468. DOI: 10.1111/jar.12308 28321970 PMC9400199

[bibr50-17446295231219301] Pytlowana A Stenfert Kroese B (2021) What are the experiences of professionals working with parents with learning disabilities? A meta-ethnography. Tizard Learning Disability Review 26(1): 14-27. DOI: 10.1108/TLDR-06-2020-0012

[bibr51-17446295231219301] Richter LM Daelmans B Lombardi J Heymann J Boo FL Behrman JR Lu C Lucas JE Perez-Escamilla R Dua T Bhutta ZA Stenberg K Gertler P Darmstadt GL (2017) Investing in the foundation of sustainable development: pathways to scale up for early childhood development. Lancet 389 (10064): 103–118. DOI: 10.1016/S0140-6736(16)31698-1 27717610 PMC5880532

[bibr52-17446295231219301] Romeo R Knapp M Scott S (2006) Economic cost of severe antisocial behaviour in children - and who pays it. British Journal of Psychiatry 188(6): 547–553. DOI: 10.1192/bjp.bp.104.007625 16738345

[bibr53-17446295231219301] Schalock RL Luckasson R Tassé MJ (2021) Intellectual disability: Definition, Diagnosis, Classification, and systems of supports (12th Edition). Washington: American Association on Intellectual and Developmental Disabilities. 10.1352/1944-7558-126.6.439 34700345

[bibr54-17446295231219301] Scheffers F Moonen X van Vugt E (2020) External sources promoting resilience in adults with intellectual disabilities: A systematic literature review. Journal of Intellectual Disabilities 26(1): 227-243*.* DOI: 10.1177/1744629520961942 32985320 PMC9016661

[bibr55-17446295231219301] Schuengel C Kef S Hodes MW Meppelder M (2017) Parents with intellectual disability. Current opinion in psychology, 15: 50-54. DOI: 10.1016/j.copsyc.2017.02.022 28813268

[bibr56-17446295231219301] Slayter EM Jensen J (2019) Parents with intellectual disabilities in the child protection system. Children and Youth Services Review 98: 297-304. DOI: 10.1016/j.childyouth.2019.01.013

[bibr57-17446295231219301] Stewart A MacIntyre G (2017) Parents with learning disabilities. Iriss Insights 37: 1-16. https://www.iriss.org.uk/resources/insights/parents-learning-disabilities

[bibr58-17446295231219301] Tarleton B Heslop P (2021) Mellow Futures–An adapted parenting programme for mothers with learning difficulties in England and Scotland. Professionals' views on the outcomes. Health & Social Care in the Community 29(5): 1275-1284. DOI: 10.1111/hsc.13164 32946142

[bibr59-17446295231219301] Tarleton B Ward L (2007) “Parenting with support”: The views and experiences of parents with intellectual disabilities. Journal of Policy and Practice in Intellectual Disabilities 4(3): 194-202. DOI: 10.1111/j.1741-1130.2007.00118.x

[bibr60-17446295231219301] Tymchuk A (1998) The importance of matching educational interventions to parent needs in child maltreatment: issues, methods, and recommendations. In: Lutzker J (ed) Handbook of Child Abuse Research and Treatment New York: Plenum press, pp.421-448.

[bibr61-17446295231219301] UNICEF UK (1989) The United Nations convention on the rights of the child. Available at: https://downloads.unicef.org.uk/wp-content/uploads/2010/05/UNCRC_PRESS200910web.pdf?_ga=2.78590034.795419542.1582474737-1972578648.1582474737 (accessed 22 September 2022)

[bibr62-17446295231219301] United Nations (2006) Convention on the Rights of Persons with Disabilities. Available at: https://www.ohchr.org/en/hrbodies/crpd/pages/conventionrightspersonswithdisabilities.aspx 10.1515/9783110208856.203 18348362

[bibr63-17446295231219301] Wade C Llewellyn G Matthews J (2008) Review of parent training interventions for parents with intellectual disability. Journal of applied research in intellectual disabilities 21(4): 351-366. DOI: 10.1111/j.1468-3148.2008.00449.x

[bibr64-17446295231219301] Wickstrom M Höglund B Larsson M , et al. (2017) Increased risk for mental illness, injuries, and violence in children born to mothers with intellectual disability: A register study in Sweden during 1999-2012. Child Abuse & Neglect 65: 124-131. DOI: 10.1016/j.chiabu.2017.01.003 28135626

[bibr65-17446295231219301] Willems DL Höglund B Larsson M Lundgren M (2007) Parenting by persons with intellectual disability: an explorative study in the Netherlands. Journal of Intellectual Disability Research 51(7): 537-544. DOI: 10.1111/j.1365-2788.2006.00924.x 17537167

[bibr66-17446295231219301] Wilson S De Vries JN Isarin J Reinders JS (2013) A systematic review of interventions to promote social support and parenting skills in parents with an intellectual disability. Child: care, health and development 40(1): 7-19. DOI: 10.1111/cch.12023 23331065

[bibr67-17446295231219301] World Health Organization, United Nations Children’s Fund and World Bank Group (2018) Nurturing care for early childhood development: A framework for helping children survive and thrive to transform health and human potential. Geneva: World Health Organization.

[bibr68-17446295231219301] Zijlstra A Sterenborg T Nieuwenhuijzen MV et al. (2023) Expectant parents with mild to borderline intellectual disabilities: Risk factors for the child’s safety. [Manuscript submitted for publication].

[bibr69-17446295231219301] Zijlstra E (2012) In the best interest of the child? A study into a decision-support tool validating asylum-seeking children’s rights from a behavioural scientific perspective*.* PhD Thesis, Rijksuniversiteit Groningen, Groningen.

